# Metabolism of Cartilage Proteoglycans in Health and Disease

**DOI:** 10.1155/2014/452315

**Published:** 2014-07-03

**Authors:** Demitrios H. Vynios

**Affiliations:** Biochemistry, Biochemical Analysis and Matrix Pathobiology Research Group, Laboratory of Biochemistry, Department of Chemistry, University of Patras, 26500 Patras, Greece

## Abstract

Cartilage proteoglycans are extracellular macromolecules with complex structure, composed of a core protein onto which a variable number of glycosaminoglycan chains are attached. Their biosynthesis at the glycosaminoglycan level involves a great number of sugar transferases well-orchestrated in Golgi apparatus. Similarly, their degradation, either extracellular or intracellular in lysosomes, involves a large number of hydrolases. A deficiency or malfunction of any of the enzymes participating in cartilage proteoglycan metabolism may lead to severe disease state. This review summarizes the findings regarding this topic.

## 1. Introduction

Proteoglycans (PGs) are a class of complex macromolecules found extracellularly in most tissues. They are composed of a core protein onto which a variable number of glycosaminoglycan (GAG) side chains are attached. According to their presence in cell and tissues, interactions with other macromolecular components, and detailed structure of the core protein, PGs are divided to different families, which has been extensively reviewed [see, e.g., [1–4]]. According to the status of the cell or the tissue, the GAG chains in a selected PG may vary in number and size.

PGs studies started almost with the beginning of the 20th century; however, they became familiar in late sixties, after the application of the pioneering extracting protocol of Sajdera and Hascall [5]. A tremendous amount of work is presented during the seventies, regarding isolation, purification, and characterization of proteoglycans from extracellular matrix in healthy and pathologic states, together with studies on their biosynthesis from cell or tissue cultures and cell-free systems. In these years and due to limitations in methodology, the studies focused mainly on tissues of high PG content, that is, cartilage, aorta, and skin. In later years, through the development of micro- and nanoanalytical techniques, PG studies were extended to other tissues poorer in PGs. By combining molecular biology techniques, the research on PGs became easier and led to novel insights into the structure and function on PGs. In the middle of the eighties, new members of PGs were clearly identified and, thus, it became evident that the term proteoglycan was not sufficient to describe the sum of heterogeneous and polydisperse macromolecules isolated from tissues. Therefore, PGs were divided to five different families, according to their structure, location, or properties. The hyalectans, having the ability to interact electrostatically with hyaluronan and link protein, the basement membrane PGs, the cell-surface PGs, the intracellular PGs, and the small leucine-rich PGs (SLRPs). The name of a selected PG was coined from a characteristic property with the suffix-can. According to this, the main proteoglycan in cartilage was termed aggrecan, since it can form aggregates with hyaluronan and link protein. Similarly, a small PG of the SLRPs family that contains two GAG chains was termed biglycan. Some exceptions are known, since some PGs are present in tissue in both PG and free core protein forms, and, thus, their name came almost exclusively from their properties, that is decorin, the PG that decorate collagen fibrils formation.

With the development of bioinformatics and the sequencing of the whole genome of various organisms, new PG members, with peculiar properties, were added.

Moreover, by sequencing PGs at DNA or protein level, characteristic protein domains were revealed, indicating their existence as multifunctional macromolecules with a great impact on cell life and behavior [6, 7], making, thus, PG research a significant field, employing scientists of different background.

Although basic research on PGs became familiar in the late sixties, the relation of PGs with specific diseases was well established much earlier. Examples are the various mucopolysaccharidoses (MPS), which constitute a family of lysosomal storage diseases characterized by deficiencies in lysosomal hydrolases responsible for the degradation of GAGs (mucopolysaccharides) [8].

## 2. Proteoglycan Structure and Populations

PGs are found in abundance in cartilage, as they represent about 10% of tissue dry weight. The typical members are shown in Table 1.

Aggrecan, formerly known as “main cartilage proteoglycan” or “large aggregating proteoglycan,” consists of a protein core of about 200 kDa molecular mass, onto which about 100 chondroitin sulfate (CS), 50 keratan sulfate (KS), and 50 *N*- and* O*-linked oligosaccharide chains are attached. Dermatan sulfate (DS) is absent from aggrecan. Through its N-terminal domain, aggrecan possesses the ability to interact with hyaluronan and link protein [6]. More than 1000 aggrecan molecules may participate in each one of these giant aggregates formed and, thus, provide a stable matrix capable of absorbing high compressive loads by water desorption and resorption. Moreover, aggregates are strong inhibitors of hydroxyapatite growth. The content and composition of aggrecan seems largely to be related with the state of tissue. For example, in aged cartilage, a decrease in the absolute amount of aggrecan is observed, together with a decrease in CS content and an increase in KS content and size.

Biglycan and decorin belong to the small and rich in leucine-repeats PG family. They possess a core protein of about 40 kDa molecular mass onto which two and one, respectively, CS/DS chains are attached. Nonglycanated forms (no GAG chains) of both PGs are also observed. These PGs help to stabilize and organize collagen fibers and, in addition, they can bind growth factors [9, 10]. Decorin can bind transforming growth factor *β* (TGF-*β*), serving as a sink to keep the growth factor sequestered in the matrix. Biglycan can bind bone morphogenetic protein-4 (BMP-4), a member of TGF-*β* family, and it plays a significant role in cartilage and bone metabolism. Both PGs amounts are substantially increased with ageing, together with increase in DS and their nonglycanated forms.

Aggrecan represents the vast majority of PGs on weight basis, although, on molecular basis, similar amounts of aggrecan, biglycan, and decorin are present in young cartilage.

## 3. Biosynthesis of Proteoglycans in Health and Disease

The protein component of PGs is synthesized by ribosomes and translocated into the lumen of the rough endoplasmic reticulum. Glycosylation of the PG occurs in the Golgi apparatus in multiple enzymatic steps. First, a special link tetrasaccharide is attached to a Ser side chain on the core protein to serve as a primer for polysaccharide growth. Then sugars are added one at a time by glycosyl transferase. The completed PG is then exported in secretory vesicles to the extracellular matrix of the tissue. Due to the complexity of the macromolecules, their biosynthesis can be examined separately, that is, biosynthesis of core proteins and biosynthesis of carbohydrate chains. Both biosyntheses have a high energy demand, especially in the case of aggrecan, and involve a plethora of different enzymes in the glycosylation step.

### 3.1. Biosynthesis of Core Proteins and* N*-Linked Oligosaccharide Side Chains

The most detailed studied biosynthesis of cartilage PGs is that of aggrecan and the results are coming from cell and tissue cultures, as well as from Swarm rat chondrosarcoma. The studies have shown that core protein biosynthesis follows the general rules of protein biosynthesis, with the modifications required for protein secretion. The addition of* N*-linked oligosaccharides close to the N-terminal end becomes very early, which are synthesized onto dolichol phosphate, as it has been supported by experiments using tunicamycin as inhibitor of N-glycosylation. Therefore, all the enzymatic machinery needed for* N*-linked oligosacchride precursor biosynthesis and processing after its incorporation onto the core protein participates during the translation step. It has also been shown that once core protein is synthesized, it spends enough time to move to the Golgi apparatus for GAG biosynthesis.

In a characteristic disease state of cartilage, osteoarthritis (OA), decreased expression of aggrecan core protein is observed, which follows changes in chondrocyte phenotype [11]. However, OA is a result of cartilage destruction through depletion of aggrecan and this is discussed below. In chondrosarcoma, the cells are continuing to synthesize aggrecan [12]; however, in other types of cancer in cartilaginous tissues, aggrecan core protein expression is highly decreased [13], possibly due to the depletion of chondrocytes. Mutations of the core protein resulted in skeletal disorders, such as nanomelia and cartilage deficiency [3].

### 3.2. Biosynthesis of Glycosaminoglycans and* O*-Linked Oligosaccharide Side Chains

**Figure 1:**
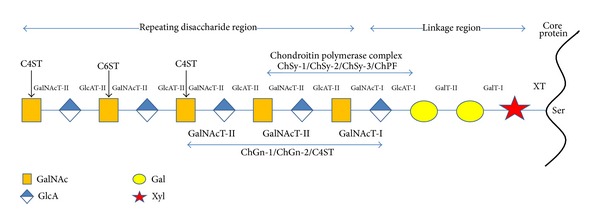
A simplified scheme for CS biosynthesis. The participation of the specific glycosyltransferases in the synthesis of the linkage region and of chondroitin chain is indicated. Chondroitin polymer is additionally modified through sulfation.

The most complex part of PGs biosynthesis is the addition of GAG chains (Figure 1). The main GAGs found in PGs, that is, CS, DS, heparan sulfate, and heparin, have as a prerequisite the presence of an* O*-linked tetrasaccharide, made of D-xylosyl, two D-galactosyl, and *β*-D-glucuronosyl residues. In the case of aggrecan, where only CS is present, the majority, if not all, of tetrasaccharides are linked to a serine residue, especially in the Ser-Gly-X sequences. The same occurs with biglycan and decorin, having CS/DS chains.

GAGs are linked to serine residues in core proteins via xylose. Xylosyl-transferase initiates the process using UDP-xylose as donor. Two isoforms of the enzyme are known in vertebrates (XT-1 and XT-2). The glycine residue in the C-terminal side of Ser contributes with its small size, and is usually followed by an acidic amino acid residue. Several PGs, especially aggrecan, contain clustered GAG attachment sites, raising the possibility that XT could act in a processive manner [14]. The extent of xylosylation of a selected PG molecule, with multiple attachment sites, varies in different cells, suggesting that xylosylation is an incomplete process. Variation in the degree of GAG substitution also might result from low levels of UDP-xylose, low activity of the XTs, or competing reactions such as Ser phosphorylation, acylation, or other forms of glycosylation, that is, addition of* O*-linked oligosaccharide side chains.

After xylose addition, the tetrasaccharide linkage region assembles by the transfer of two galactose residues catalyzed by unique members of the *β*1–4 galactosyl-, *β*1–3 galactosyl-, and *β*1–3 glucuronyl-transferase families of enzymes, termed GalT-I, GalT-II, and GlcAT-I, respectively [15]. This intermediate can undergo phosphorylation at the C-2 position of xylose and sulfation of the galactose residues. In general, phosphorylation and sulfation occur substoichiometrically. The lack of chain specificity for phosphorylation would seem to exclude it as a signal for controlling composition. Phosphorylation may be transient, suggesting a role in processing or sorting. Galactose sulfation is found only in CS, but its role in chain initiation, polymerization, or turnover remains unclear.

Once the linkage tetrasaccharide is formed, the biosynthesis of the entire GAG can now start through the addition of* N*-acetyl-*β*-D-galactosamine (D-GalNAc) linked by a *β*1–4 glycosidic bond. This process, that is, addition of *β*4-D-GalNAc or *α*4-D-GlcNAc, seems to be manifested at the level of enzyme recognition of the polypeptide substrate [15]. Two enzymes possessing* N*-acetyl-D-galactosaminyl-transferase (GalNAcT) activity exist, GalNAcT-I and GalNACT-II, which are involved in chain initiation, as well as in chain polymerization. Additional enzymatic activity participates in chain elongation, which is usually referred to as chondroitin synthase, because of its bifunctional role in the polymerization process; that is, it is acting as both *β*-D-glucuronyl-transferase (GlcAT-II) and *β*-*N*-acetyl-D-galactosaminyl-transferase (GalNAcT-II). Studies that took place during the last decade have identified and cloned six homologous glycosyl-transferases responsible for biosynthesis of chondroitin repeating units [15]. According to their enzymatic activity* in vitro*, the members of this family were designated as ChSy-1 (chondroitin synthase-1), ChSy-2, ChSy-3, ChPF (chondroitin-polymerizing factor), and ChGn-1 (chondroitin GalNAc transferase-1) and ChGn-2, respectively. The first three enzymes, ChSy-1, ChSy-2, and ChSy-3, are those possessing dual glycosyl-transferase activities, GlcAT-II and GalNAcT-II, but cannot polymerize chondroitin chains by themselves [16–19]. However, coexpression of any two of four proteins, ChSy-1, ChSy-2, ChSy-3, and ChPF, that has only marginal GalNAcT-II activity each [17, 20], markedly increases the ability of GlcAT-II and GalNAcT-II activities to synthesize chondroitin polymer. This indicates the importance of the chondroitin-polymerizing factor, an enzyme lacking independent activity but acting together with the transferases to enhance the formation of polymers. On the other hand, the latter two enzymes, ChGn-1 and ChGn-2, possess both GalNAcT-I and GalNAcT-II activities and, therefore, are thought to catalyze chain initiation and elongation of chondroitin [15]. Biosynthesis of chondroitin chain onto the core protein seems to occur much later than translation.

The newly synthesized chondroitin chain is subjected to modifications, such as epimerization and sulfation. Both are the very late stage in PG biosynthesis and occur at the same time and just before PG secretion. Epimerization is highly dependent to sulfation; however, in the case of aggrecan, it does not occur at all. Sulfation is a complex process, and multiple sulfotransferases are involved in 4-*O* sulfation and 6-*O* sulfation of GalNAc residues. Four 4-*O*-sulfotransferases have been characterized in mammals [21–25] and termed C4ST-1 (chondroitin 4-*O*-sulfotransferase-1), C4ST-2, C4ST-3, and D4ST-1 (dermatan 4-*O*-sulfotransferase-1). The first three preferably catalyze the 4-*O*-sulfation of GalNAc residues in CS, whereas the latter is responsible for the 4-*O*-sulfation of GalNAc residues next to iduronate in DS. Sulfation at C-6 position of GalNAc residues in CS is mediated by C6ST-1 (chondroitin 6-*O*-sulfotransferase-1) [26]. It is questionable the presence of a sulfotransferase responsible for 6-*O*-sulfation in DS. All sulfotransferases use activated sulfate (PAPS; 3′-phosphoadenosine-5′-phosphosulfate) as a high-energy donor. In humans, sulfation occurs mainly at the C-6 of GalNAc, and only a small number of residues remain unsulfated. In other vertebrates, sulfation occurs mainly at C-4 of GalNAc. In all animal species, sulfation at C-6 of GalNAc increases with aging. Additional enzymes exist for epimerization of glucuronic acid to iduronic acid in DS, sulfation at the C-2 position of the uronic acids, but none of them act on aggrecan. In species other than human, additional sulfotransferases exist and unusual patterns of sulfation are observed.

In cell or tissue culture experiments in the presence of *β*-D-xyloside, a precursor of GAG biosynthesis, the PGs produced contain fewer and shorter CS/DS chains, with decreased sulfation, suggesting that the number of putative chain initiation sites controls both size and sulfation of GAGs [27]. Evidence is also presented indicating that ChGn-1 together with C4ST-II regulate the initiation of CS synthesis and, thus, control the number of CS chains attached to core protein [28]. Moreover, chain elongation is controlled by a cooperation between C4ST-1 and ChGn-2 [29]. It should also be noted that, in cell cultures in the presence of monensin, an ionophore that impairs Golgi apparatus, an accumulation of core proteins within the cells is observed, suggesting the functional role of Golgi apparatus in PGs biosynthesis [30]. However, monensin seems not to affect GalNAc-6-sulfation, but mainly epimerization of glucuronate and GalNAc-4-sulfation, providing evidence on the organization of transferases in Golgi apparatus [31].

The enzymes involved in chondroitin polymerization are indispensable for life. However, it has been revealed that knockout mice for either of* ChSy-1*,* ChPF,* or* ChGn-1* are viable and fertile, although they do synthesize reduced CS amounts with an imbalance in its sulfation [32–35]. Due to the decreased CS content and sulfation, especially in cartilaginous tissues, the animals display skeletal abnormalities including chondrodysplasia, decreased bone density, and digit-patterning defects. In addition, knockdown of* ChSy-1* in zebrafish embryos lead to skeletal anomalies and craniofacial and inner ear dysmorphogenesis. In human, mutations in* CHSY1* have been identified in syndromic recessive preaxial brachydactylies, mainly characterized by limb malformations, short stature, and hearing loss [36, 37]. These findings suggest the essential regulatory roles of ChSy-1 in skeletal development. Moreover, a mutation of C6ST-1 abolishes its activity and results in severe human chondrodysplasia with progressive spinal involvement. Mutation of PAPS synthetase results in skeletal abnormalities, known as brachymorphism [3].

Ehlers-Danlos syndrome (progeroid type) is another disorder characterized by deficiencies in biosynthetic enzymes related to glycosylation of PGs. Decorin is partially deficient since some molecules are secreted from cells as free proteins without containing the GAG chain. This is because the enzymes GalT-I and GalT-II have very low activity compared to normal [14].

In cancer of cartilaginous tissues, many changes at the expression level of almost all enzymes involved in CS/DS biosynthesis are observed [38]. Little evidence has been provided regarding the cellular pool responsible for the altered biosynthesis, which suggests that the malignant cells penetrating cartilage are responsible for at least some of these changes.

The biosynthesis of KS on aggrecan (skeletal KS or KSII) follows a different scheme. KS is linked through D-GalNAc to Ser or Thr of the core protein. Then a poly-*N*-acetyllactosamine chain follows which is sulfated somewhat randomly. At least two classes of sulfotransferases, one or more GlcNAc-6-*O*-sulfotransferases, and one galactose-6-*O*-sulfotransferase catalyze the sulfation reactions [39]. Similarly as above, these enzymes use PAPS as a high-energy donor. It seems that GlcNAc 6-*O* sulfation occurs only on the nonreducing terminal GlcNAc residue, whereas sulfation of galactose residues takes place on nonreducing terminal and internal galactose residues, with a preference for galactose units adjacent to a sulfated GlcNAc [39]. Sulfation of a nonreducing terminal galactose residue blocks further elongation of the chain, providing a potential mechanism for controlling chain length. Another mechanism for controlling KS chain elongation is the addition of a sialic acid residue. In some cases, KS seems to contain a small branch early terminated by sialic acid addition. The content and the size of KS in aggrecan molecule are highly affected by aging. Aged cartilage contains aggrecan substituted with more KS chains of higher molecular size [40, 41]. It seems that chain elongation control is affected by aging. KSII is not present in all animal species, but only in these containing an aggrecan sequence that possesses a specific KS-binding region, characterized by the presence of the amino acid sequence Glu-Glu/Leu-Pro-Phe-Pro-Ser [39]. KS biosynthesis is often markedly altered in response to metabolic, pathologic, or developmental changes in tissues.

## 4. Degradation of Proteoglycans in Health and Disease

**Figure 2:**
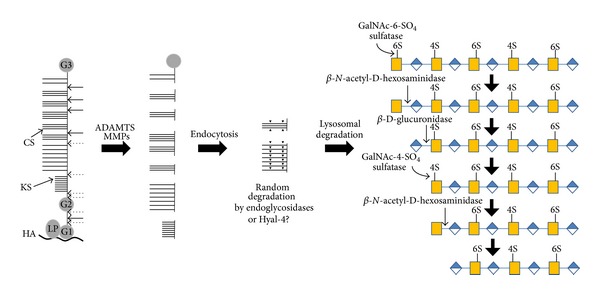
A simplified scheme for aggrecan degradation. Aggrecan is initially degraded by extracellular proteases, mainly ADAMTS and MMPs; the various fragments are endocytosed and the CS-containing peptides are further processed by specific glycosidases and sulfatases. Only the initial endolysosomal degradation steps are shown.

Similarly as biosynthesis, degradation of PGs is a process catalyzed by different enzymes for core proteins and GAG chains. Initial degradation of PGs occurs extracellularly, due to their molecular size, and especially that of aggrecan, that does not permit endocytosis of the whole molecule. Various metalloproteinases, like MMPs (matrix metalloproteinases), ADAMTSs (A disintegrin and metalloproteinase with thrombospondin motif), and cysteine proteinases, are usually involved [42, 43]. Degradation also occurred by free radicals and mechanical stress. The fragments are endocytosed and subjected to further degradation in lysosomes (Figure 2). In various types of arthritis, increased extracellular degradation of aggrecan is observed due to increased MMPs and ADAMTS activity [42], as well as increased and secreted cathepsins activity [44].

### 4.1. Degradation of Core Proteins

Aggrecan core protein is sensitive to proteolysis at numerous sites along its length. Cleavage at any of these sites results in the removal of a part (of different size, depending on the cleavage site) of CS chains and, thus, affecting its negative charge. Cleavage of aggrecan in the interglobular domain (IGD) between the N-terminal G1 and G2 globular domains is of greatest importance, as this releases the whole GAG bearing region of aggrecan from the cartilage matrix and so abrogates the function of the molecule [42, 45].

In healthy cartilage, the extracellular proteolytic enzymes are present in their latent forms. Moreover, the presence of specific tissue inhibitors of metalloproteinases (TIMPs) has a negative effect on their activity, although* in vitro* studies suggest that MMPs participate in normal turnover of aggrecan [42, 46]. It should be noticed that a member of TIMPs family, TIMP-3, possesses the ability to inhibit ADAMTS-4 and -5 [42]. As TIMP-3 can inhibit both MMPs and ADAMTSs, it is a central regulator of normal turnover of cartilage macromolecules. Nevertheless, in healthy cartilage, about 10–15% of the aggrecan molecules appeared to be free, in a nonaggregating state in the tissue, because they lack their N-terminal region. The presence of fragmented aggrecan is explained by the normal recycling of extracellular macromolecules. It has been proposed that m-calpain, a calcium-dependent cysteine proteinase, may be responsible for extracellular cleavage of aggrecan in CS1 region [43]. Aggrecan fragments are internalized to the cells and degraded further in lysosomes by their proteolytic machinery, the cathepsins. Cathepsins are present in lysosomes in their latent forms and are activated through their interaction with GAGs [44]. The CS chains are also degraded in lysosomes and their degradation is discussed below.

Among the pathologic states of cartilage, of particular importance are the various types of arthritis, OA being the most important. Degradation of aggrecan is an early event in the development of OA and a lot of research has been done to identify the enzymes responsible. It was initially thought that MMPs were the primary aggrecan-degrading enzymes in OA, since several MMPs, that is, MMP-1, -2, -3, -7, -8, -9, and -13, were found to be able to cleave the Asn341~Phe342 bond in the aggrecan IGD, the first one being MMP-3 [47]. However, evidence was presented revealing that the majority of aggrecan fragments present in the synovial fluid of OA patients were cleaved not at the MMP-sensitive Asn341~Phe342 bond, but at the Glu373~Ala374 bond in the IGD. Moreover, this cleavage was not blocked by the tissue inhibitors of MMPs, TIMP-1, TIMP-2, or synthetic MMP inhibitors [48], indicating that enzymes other than MMPs are responsible for the activity. These findings led to the identification of ADAMTS as the main enzymes attacking aggrecan in at least the early events of OA. ADAMTS, like all metalloproteinases, are synthesized by the cells in latent forms that require activation to express their activity. Activation proceeds mainly through the action of members of the family of proprotein convertases [49].

Activated ADAMTS-1, -4, -5, -8, -9, -15, -16, and -18 can degrade aggrecan* in vitro *[50–53], but ADAMTS-5 is the most active enzyme* in vitro*, followed by ADAMTS-4 [54], and there is some evidence suggesting that ADAMTS-4 contributes to cartilage degradation. ADAMTS-4 and ADAMTS-5 are, thus, considered to be the major enzymes responsible for pathological cleavage of aggrecan in OA, ADAMTS-5 having a pivotal role [55]. However, since aggrecan cleavage at the MMPs sensitive bond is also detectable in OA cartilage [56], MMPs may also act on aggrecan later in the progression of disease. ADAMTS can also cleave biglycan [57] and, thus, may affect overall tissue organization, contributing even more in cartilage destruction.

It should also be noticed that secretion of lysosomal cathepsins was observed in OA. As the pH of OA cartilage is lower than normal [58], cathepsins largely participate in proteoglycan degradation extracellularly.

There is also some evidence that aggrecan depletion that occurred in cartilaginous tissues in cancer progression is mediated through the action of ADAMTS, and especially ADAMTS-5 [59]. MMPs have also shown that they participate in cartilage destruction in cancer [60], but it is not clear whether they attack aggrecan.

### 4.2. Degradation of Glycosaminoglycans

The CS/DS-containing peptides once they are endocytosed they are transferred to lysosome when their protein parts are further degraded by acidic proteolytic enzymes and their carbohydrate parts by specific glycosidases. The degradation process of GAGs is highly ordered and employs endo- and exoglycosidases and sulfatases, sometimes aided by noncatalytic proteins. Although the presence of lysosomal hyaluronidase, an enzyme degrading hyaluronan and, to a lesser extent, CS, is observed in cartilage extracellular matrix, no evidence has yet been presented for its involvement in extracellular degradation of CS. Hyaluronidase, an endo-*β*-*N*-acetyl-D-glucosaminidase, seems to be the first glycosidase that hydrolyzes CS on CS/DS-containing peptides to large oligosaccharides. There exist also enzymes, such as endo-*β*-D-glucuronidases or endo-*β*-D-hexosaminidases, cleaving CS/DS at a few specific sites. Alternatively, the specific hyaluronidase for CS, Hyal-4, may be responsible for its initial cleavage [61, 62]. However, the exact mechanism for initial CS/DS degradation is not yet clear. Then, specific sulfatases (GalNAc-6-SO_4_ sulfatase and GalNAc-4-SO_4_ sulfatase) remove the sulfate ester groups and the exoglycosidases *β*-D-glucuronidase or *α*-L-iduronidase and *β*-*N*-acetyl-D-galactosaminidase (or *β*-*N*-acetyl-D-hexosaminidase), acting in parallel, cleave the glycosidic linkage of terminal sugars from the nonreducing end of the oligosaccharides to liberate the monosaccharides. The tetrasaccharide linkage region is degraded by the appropriate specific glycosidases.

On the other hand, the mammalian cells do not have an endoglycosidase to cleave KS, possibly because its molecular size is small compared to that of CS/DS. Therefore, the KS-containing peptides are hydrolyzed in lysosomes by the sequential action of sulfatases and exoglycosidases. Galactose-6-SO_4_ sulfatase is the same enzyme that desulfates GalNAc-6-SO_4_ in CS degradation. The exoglycosidases involved are *β*-galactosidase and *β*-*N*-acetyl-D-hexosaminidase.

As it is mentioned in the Introduction, defective degradation of GAGs in lysosomes is the origin of the various mucopolysaccharidoses [63]. The absence or the malfunction of a single lysosomal hydrolase leads to the accumulation of its substrate as undegraded fragments in tissues and the appearance of related fragments in urine. The result is permanent, progressive cellular damage which affects appearance, physical abilities, organ and system functioning, and, in most cases, mental development. The pathology likely depends on the cell type and the cellular balance of synthesis and turnover rates. Hurler syndrome, Hurler-Scheie syndrome, and Scheie syndrome have a defective *α*-iduronidase, Hunter syndrome a defective iduronate sulfatase, Morquio syndrome A a defective GalNAc-6-SO_4_ sulfatase, Morquio syndrome B a defective *β*-galactosidase, Maroteaux-Lamy syndrome a defective N-acetylgalactosamine-4-SO_4_ sulfatase, and Sly syndrome a defective *β*-D-glucuronidase. The first four syndromes accumulate degradation products of DS (and of heparan sulfate), Morquio syndromes of KS and chondroitin-6-sulfate (A) and of KS (B), Maroteaux-Lamy syndrome of DS and Sly syndrome of DS/DS (and of heparan sulfate).

A very rare human disorder is the multiple sulfatase deficiency that affects all GAGs degradation.

## 5. Concluding Remarks

Both biosynthesis and degradation of cartilage PGs are processes involving multiple enzymes. There is increasing evidence that a deficiency or malfunction of any of the enzymes participating in any of these processes may lead to severe cellular or organ malfunction or damage. The biosynthesis of deficient PGs may affect their charge density or their interactions with other extracellular components, and it affects cartilage structure and properties. The deficient degradation of PGs may accumulate limited degradation products within the cell with harmful effects to the organism. Extended studies are performed to understand the molecular mechanisms involved and synthetic or natural drugs are applied as therapeutic tools.

For the most common cartilage disease, OA, current treatments are symptomatic and limited by side effects or lack of efficacy, and despite using them, many people with OA still have significant symptoms. New approaches to targeting pathology offer hope of new analgesic options and, for the first time, structure modification may be possible by treating a noncartilage target, the subchondral bone. In addition, gene transfer approaches allow for a long-term and site-specific presence of a therapeutic agent to reequilibrate the metabolic balance in OA cartilage and may consequently be suited to treat this slow and irreversible disorder. In an interesting clinical trial, the chondrocytes of patients have been modified to produce the transforming growth factor *β*1 via intra-articular injection, showing a dose-dependent trend toward efficacy. However, the distinct stages of OA need to be respected in individual gene therapy strategies, and molecular therapy appears to be most effective for early OA.

## Figures and Tables

**Table 1:** Structural proteoglycans of cartilage.

Proteoglycan	Molecular mass of core protein	Number of GAG chains	Molecular mass of GAG chain
Aggrecan	208–220	~100	~14
Decorin	36	1	~28
Biglycan	38	1-2	~28

## Abbreviations

ADAMTS: A disintegrin and metalloproteinase with thrombospondin motif
BMP-4: Bone morphogenetic protein-4
C4ST-1: Chondroitin 4-*O*-sulfotransferase
C6ST-1: Chondroitin 6-*O*-sulfotransferase-1
ChGn-1: Chondroitin GalNAc transferase-1
ChPF: Chondroitin-polymerizing factor
ChSy-1: Chondroitin synthase
CS: Chondroitin sulfate
D4ST-1: Dermatan 4-*O*-sulfotransferase-1
D-GalNAc: * N*-acetyl-*β*-D-galactosamine
DS: Dermatan sulfate
GAG: Glycosaminoglycan
GalNAcT: * N*-acetyl-D-galactosaminyl-transferase
GalT: Galactosyl-transferase
GlcAT: Glucuronyl-transferase
IGD: Interglobular domain of aggrecan
KS: Keratan sulfate
MMP: Matrix metalloproteinase
MPS: Mucopolysaccharidoses
OA: Osteoarthritis
PAPS: 3′-Phosphoadenosine-5′-phosphosulfate
PG: Proteoglycan
SLRPs: Small leucine-rich PGs
TGF-*β*: Transforming growth factor-*β*
TIMP: Tissue inhibitors of MMPs
XT: Xylosyl-transferase.
